# The impact of National Containment Measures on a Pediatric Italian regional Hub for COVID-19, an observational study

**DOI:** 10.1186/s13052-021-01081-w

**Published:** 2021-06-02

**Authors:** Francesca Crea, Filippo Maria Panfili, Maria Elisa Amodeo, Danilo Fintini, Francesco Paolo Rossi, Italo Trenta, Alessandra Menichella, Chiara Ossella, Andrea Deidda, Roberta Lidano, Giulia Macchiarulo, Caterina Lambiase, Maria Antonietta Barbieri, Massimiliano Raponi

**Affiliations:** 1 Pediatric Emergency Department, Bambino Gesù Children’s Hospital, IRCCS, Piazza di Sant’Onofrio, 4, 00165 Rome, Italy; 2 University of Rome Tor Vergata, Rome, Italy; 3 Endocrinology Unit, University-Hospital Pediatric Department (DPUO), Bambino Gesù Children’s Hospital, IRCCS, Rome, Italy; 4 Occupational Medicine/Health Technology Assessment and Safety Research Unit, Clinical-Technological Innovations Research Area, Bambino Gesù Children’s Hospital, IRCSS, Rome, Italy; 5 Health Directorate, Bambino Gesù Children’s Hospital, Istituto di Ricovero e Cura a Carattere Scientifico, Rome, Italy

**Keywords:** COVID-19, SARS-CoV 2, Pediatric, Emergency department, Children

## Abstract

**Background:**

Numerous studies described the epidemiological link and main clinical features of pediatric COVID-19, during the first pandemic period. Our study encompasses several different phases since the National Lockdown in Italy. The primary outcome is (I) to analyze the prevalence of positive NST (Nasopharyngeal Swab Test) among the largest Italian Pediatric cohort admitted to a single regional PED Hub for COVID-19 during an eight-month period. Secondary outcomes are: (II) the description of trend of admissions in our PED and (III) the categorization of the positive patients according to clinical manifestations and epidemiological link.

**Methods:**

We described 316 patients with a positive NST for SARS-CoV2, on a total of 5001 nasopharyngeal swabs performed among 13,171 admissions at our PED, over a period starting from March 17th, 2020 to December 1st, 2020. Age, epidemiological link, clinical features and hospitalizations were analyzed according to different lockdown phases. Data were collected anonymously from electronic records and analyzed using SPSS 22.00 statistics software (Chicago, IL).

**Results:**

Thirty-six percent of total admissions have been tested. During the post lockdown period, we performed the highest percentage of NST (Nasopharyngeal Swab Test) 49.7%, and among them 7.9% were positive. The prevalence of infection during a 10-month period was 2.3%. Mean age was 6.5 years old. Familial Link accounted for the 67.7% of infection, while Extrafamilial and Unknown link accounted for 17 and 14.9%, respectively. Familial link is predominant during all phases. Seventeen patients showed an intra-scholastic link, and the highest prevalence was observed in the 7–10 years age group, with a prevalence of 12.8% (5 patients). Fever was the most frequent symptom (66%), in particular among preschooler children aged 0–6 years (71.9%). Older children were more frequently symptomatic. Seven patients were admitted with MIS-C diagnosis.

**Conclusions:**

Different levels of containment measures caused important changes in number of positive NST for SARS-CoV2. Familial link was predominant in our cohort, during all phases of Lockdown. The risk of being infected at home is four time greater than the risk of being infected from an extra familial individual. Further studies are needed to evaluate the clear impact of intra-scholastic link. The constant improvement in knowledge on onset symptoms and risk factor for SARS-CoV2 infection and its complications (e.g. MIS-C), can impact on number of hospitalizations, ICU admissions and early management.

## Introduction

### Background

On March 11th, 2020 the World Health Organization (WHO) characterized COVID-19, the disease caused by SARS-Cov2, the seventh of coronavirus family, as a pandemic.

Globally, from January 3rd, 2020 to December 9th, 2020, there have been 67,530,912 confirmed cases of COVID-19 and 1,545,140 deaths, reported from WHO [1].

Clinical conditions vary from asymptomatic patients to patients presenting with symptoms of upper respiratory tract infection to moderate/severe manifestation such as pneumonia, severe acute respiratory distress syndrome (ARDS), multi-organ failure (MOF) and even death.

Children seemed, in the first phase of pandemic, to be less easily infected compared to other demographic groups of population. In addition, children showed a milder symptomatology and less complications than adults, with the exception of high-risk-categories. Infection seemed less aggressive compared to adult cohorts and fatality rate in children and adolescents was lower [2–4].

In Italy, the first European country struck by the SARS-Cov2, from January 3rd to December 9th, 2020, there have been 1,742,557 confirmed cases of SARS-Cov2 infection with 60,606 deaths [1].

According to a recent Italian study enrolling 3836 pediatric patients, clinical presentation was mild in 32.4% of cases and severe in 4.3%, particularly in pre-scholar children ≤6 years old; 4 deaths occurred. Among the cohort 13% patients were hospitalized and 3.5% were admitted in ICU. Higher risk of severity was associated with pre-existing underlying medical conditions [5].

The Italian National Health Institute (NHI) updated to December 2020 accounted about 1,624,269 affected cases, resulted positive to RT-PCR at nasopharyngeal swab test (NST), from the beginning of pandemic, including 59,044 cases of 0–9 years of age (3.6% of total affected people) with 7 deaths and 135,691 of 10–19 years of age (8.4%) with 5 deaths [6].

Comparing these data with the previous report of NHI updated to June 2020, it emerges an increase of 4–4.7 folds in the cumulative incidence respectively of group of 0–9 years old and the group of 10–19 years old (0.9% VS 3.6 and 1.8% VS 8.4% of the total affected people of the selected period respectively) [7].

### Aim of the study

The primary outcome of our study is (I) to analyze the prevalence of positive NST (Nasopharyngeal Swab Test) among the largest Italian Pediatric cohort admitted to a single regional PED Hub for COVID-19 during an eight-month period. We described the trend of infection, epidemiological links and main clinical features, according to the age of patients and different phases of Lock-down, exploring the possible influence of different Lock-down phases on these clinical and epidemiological variables. Secondary outcome are: (II) the description of trend of admissions in our PED and (III) the categorization of the positive patients according to clinical manifestations and epidemiological link.

## Methods

### Study population

In this retrospective study we evaluated the principal features of 316 pediatric patients with SARS-Cov2 infection, admitted in our PED in a period ranging from March 17th, 2020 (date on which has been performed the first test for SARS-Cov2 in our PED, and our PED become the regional Hub for COVID-19 disease) to December 1st, 2020. Out of a total of 13,703 admissions, 5001 children have been tested for SARS-Cov2 infection. All patients have been tested with a RT-PCR for SARS-Cov2 on NST.

Since March 2020 our protocol for the detection of suspected cases has been changed several times, due to the progressive increase in the numbers of the involved countries, the constant change of the leading signs and symptoms at the onset of the disease (especially in pediatric setting) and the difficulties in tracing the contacts of infected people. Currently, every patient and caregiver who is admitted at our PED, is screened with a rapid “Evaluation Form”, which includes epidemiological links and clinical manifestations (e.g., fever, respiratory symptoms, loss of smell/loss of taste and gastrointestinal symptoms). “Possible” or “probable” case, according to the eCDC [8] has to be isolated and assisted by medical staff wearing adequate “Droplet” and “Contact” Personal Protective Equipment (PPE), according to the WHO [9] and CDC [10] guidelines. Subsequently a Nasopharyngeal Swab Test is obtained from the “suspect case” and sent to our Microbiology Laboratory to perform a RT-PCR for SARS-Cov2.

Patients were divided in 5 different age groups: 0–6 years old, 7–10 years old, 11–15 years old and 16–18 years old.

We have also clustered our patients by the different phases since the First National Lockdown, as shown in Table 1.

**Table 1:** Italian Containment Measures

**Lockdown** From March 9th, 2020 to April 25th, 2020	Since March 5th, 2020, Universities and Italian schools have been closed. On March 9th, the first national Lockdown in Europe was declared in Italy. The Italian Ministry of Health recommended, in case of fever or respiratory symptoms, to avoid direct access to Emergency Departments (ED), in favor of phone consultation and home care for patients with mild or moderate diseases [11].
**Phase 2** From April 26th, 2020 to June 10th, 2020	On April 26th, 2020 started a progressive reduction of the containment measures, resuming manufacturing, construction, wholesale activities, and allowing the opening of public parks, visits to relatives within the regional territory and outdoor physical activity [12].
**Phase 3** From June 11th, 2020 to 13th September, 2020	From June 11th the Italian Government, comforted by the decline in the numbers of infected people, decided to reopen of gyms, barbers, beauty salons, bars, restaurants and bathing establishments. During summer the Italian Government gave the go-ahead to the relaunching of contact sports, summer camps for children, clubs, ballrooms and finally the possibility of moving between different regions. Moreover, the use of protective face masks was non-mandatory in public, except in crowded areas and stores [13].
**Phase 4** From September 14th,2020 to November 2nd, 2020	On September 14th, 2020 the Italian Containment Measures became less stringent, schools and university were reopened, regardless of criticism of a part of public opinion, for the risk of being infected during the lessons and on public transports due to their use over the maximum capacity [14–16].
**New Containment Measures** From November 3rd, 2020 to December 1st, 2020	Due to a new alarming increase of SARS-Cov2 infection, probably as a result of the excessive relaxation of preventing measures, Italian Prime Minister announced, on November 3rd, 2020 the New Containment Measures which provided the introduction of three different areas in the country, based on different restriction measures, from the red areas (maximum risk) to the yellow areas (lower risk) [17, 18].

Furthermore, all patients considered were analyzed for epidemiological Link: the epidemiological clusters have been individuated according to the eCDC guidelines [19]. The Familial link requires a close contact, with a family member or a cohabiting person, with a previous positive NST. The Extra-familial link requires a close contact to a person with a positive NST, outside the family (e.g., school, gym, summer camp etc.…). Unknown link refers to those cases where it was not possible to trace a clear contact with an infected person.

We have also evaluated our patients for main clinical manifestations, imaging (X-ray Chest and abdominal UltraSound), Blood tests and high-risk category.

### Data collection and laboratory testing

All NST have been performed to detect SARS-CoV2 RNA with RT-PCR according to the eCDC guidelines and tested envelope protein gene (E), nucleocapsid protein gene (N), and RNA dependent RNA polymerase gene (RdRp) [19]. All data in this study have been collected anonymously from electronic medical records. All information has been collected during the ED assessment. Hospital Ethical Committee approval and informed consents from parents/caregivers have been obtained.

### Statistical analysis

Data were analyzed using SPSS 22.00 statistics software (Chicago, IL). Continuous variables were analyzed using one way ANOVA and post hoc analysis with Bonferroni’s and Tukey Methods. Categorical variables were reported as absolute numbers or percentage and compared with χ2, Fisher exact test with confidence intervals of 95% and significance level set at 0.05. Standard deviations or median and interquartile ranges have been used to express continuous variables.

## Results

### Epidemiological trend of SARS-Cov2 infection in our pediatric emergency department

**Fig. 1:**
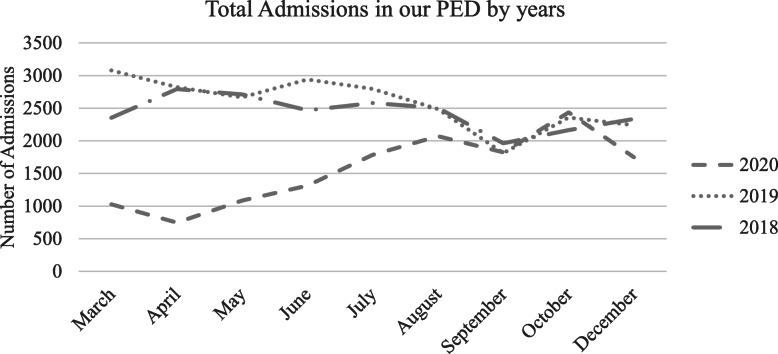
This figure shows the trend of admissions in our PED in three different years. PED = pediatric emergency department

In our PED of Bambino Gesù Children’s Hospital situated in Palidoro, Rome (Italy), the first NST for SARS-Cov2 was performed on March 17th, 2020, while the first positive test was detected on March 20th, 2020. In Fig. 1 is shown the number of visits at our hospital in from 2018 to 2020. During the covered period of our study out of a total of 13.703 admissions, 5001 children have been tested for SARS-CoV2 (36,5%). Among them 316 resulted positive for SARS-CoV2 infection.

Mean age of total patients is 6.5 years old and median age is 5 years old. It is noteworthy that during the first National Lockdown mean age and median age tended to be higher than any other phase (mean age = 9.8 and median age = 12.1, SD 6.2, IQR = 1.0–15.6), during phase 3 mean age and median age were lower (mean age = 4.8 and median age = 1.2, SD = 6.0, IQR = 0.1–17.7), although not statistically significant (*p* > 0.05).

**Fig. 2:**
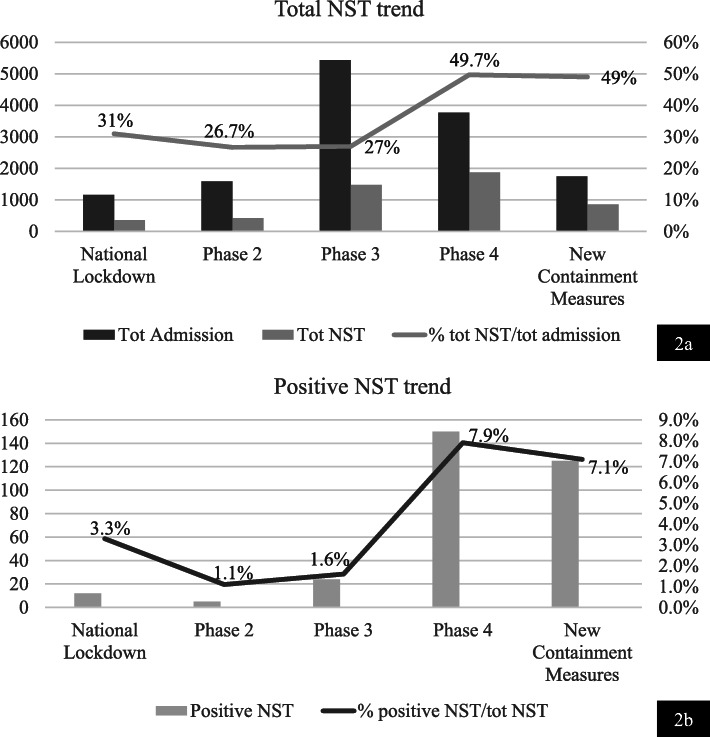
**a** In this figure it is shown the total number of NST per Lockdown phase. Total admission (N): National Lockdown: 1157, Phase 2: 1586, Phase 3: 5435, Phase 4: 3775, New Containment Measures: 1750. Total NST (N): National Lockdown: 358, Phase 2: 425, Phase 3: 1478, Phase 4: 1879, New Containment Measures: 860. Tot Admissions = total admissions, tot NST = total nasopharyngeal swab test, N = number of patients, % = percentage. We have found *P* < 0.05 for all the variables and trends through categories (ANOVA with post HOC analysis). **b** In this figure it is shown the trend of NST effectuated during the different Lockdown phases. Positive NST (N): National Lockdown: 12, Phase 2: 5, Phase 3: 24, Phase 4: 24, New Containment Measures: 125. N = number of patients. NST = Nasopharyngeal Swab Test. % = percentage. We have found *P* < 0.05 for all the variables and trends through categories (ANOVA with post HOC analysis)

In Fig. 2a are reported total admissions to our PED divided by different phases. We observed that the percentage of total NST performed on total admissions is higher since the phase 4 with a maximum of 49.7% (Fig. 2a). Interestingly also the percentage of positive NST on the total of NST is higher during the phase 4 and the New Containment Measures phases, with a maximum of 7.9% (150 positive NST) (Fig. 2b). This finding confirms the higher diffusion of infection in the pediatric population during the phase 4, along with the higher number of NST performed.

We evaluated, then, (Table 2) the incidence of positive NST among four different age groups divided by the Lockdown Phases. The age group with the highest number of positive NST is the group of preschoolers, between 0 and 6 years. The second group by number of positive NST is the group between 11 and 15 years old, with the highest number of positive NST during the phase 4. Noteworthy the age group 7–10 years old, including school-age children, has a low number of positive NST, compared to the previous two groups, even after the phase 4 and the reopening of schools. The group of young adults, 16–18 has the lower finding of positive NST, but as a pediatric hospital, the majority of admissions are under 15 years old. These results were statistically significant.

**Table 2:** Children positive for SARS-CoV2 during the different phases of Lockdown divided in age groups

	Age groups by Lockdown Phases						
		Lockdown Phases					
		National Lockdown	Phase 2	Phase 3	Phase 4	New Containment Measures	Total
**Age Groups**	**0–6** yrs. (N)	4 (33.3%)	2 (40%)	18 (75%)	79 (52.7%)	68 (54.4%)	171 (54.1%)
	**7–10** yrs. (N)	0 (0%)	2 (40%)	0 (0%)	17 (11.3%)	20 (16%)	39 (12.3%)
	**11–15** yrs. (N)	5 (41.7%)	1 (20%)	2 (8.3%)	41 (27.3%)	31 (24.8%)	80 (25.3%)
	**16–18** yrs. (N)	3 (25%)	0 (0%)	4 (16.7%)	13 (8.7%)	6 (4.8%)	26 (8.2%)

*P* < 0.05 for all the variables and trends through categories

### Epidemiological links

In Table 3 we evaluated the trend of positive NST for Epidemiological Link Clusters by Age Groups. The epidemiological clusters have been individuated according to the eCDC guidelines. Familial link was predominant in all age categories and reported in 214 children on the total of 316 (68%). Evaluating patients for the epidemiological link, we found, among the Familial Link exposure, the 0–6 years old group (62.1%) accounted for the majority of cases. Among the Extra-Familial Link exposure 11–15 years old group (40%) accounted for the majority of cases, immediately followed by the 0–6 years old group (36.4%). Finally, among the Unknown Link exposure we observed the same number of patients, 18 (38.3%) in 0–6- and 11–15 years old age groups. We have found statistically significant differences in all the variables and trends through categories.

**Table 3:** Children with NST positive for SARS-CoV2 divided for Epidemiological Link Clusters by Age Group

	Positive NST trend: Epidemiological Link by Age Groups					
	Age Groups					
		0–6 yrs	7–10 yrs	11–15 yrs	16–18 yrs	Total
**Epidemiological Link Clusters**	**Familial** (N)	133 (62.1%)	27 (12.6%)	40 (18.7%)	14 (6.5%)	214 (67,7%)
	**Extra-Familial** (N)	20 (36.4%)	8 (14.5%)	22 (40%)	5 (9.1%)	55 (17,4%)
	**Unknown** (N)	18 (38.3%)	4 (8.5%)	18 (38.3%)	7 (14.9%)	47 (14,9%)
	**Total**	171 (54,1%)	39 (12,3%)	80 (25,3%)	26 (8,2%)	316 (100%)

*P* < 0.05 for all the variables and trends through categories (ANOVA with post HOC analysis)

As it can be observed in Table 4, we also evaluated the trend of positive NST for Epidemiological Links Clusters by Age Groups. Familial link was predominant during all phases of Lockdown, with a predominance during the first lockdown and phase 2. Extra-Familial link and Unknown Link are most represented in the initial part of the New Containment Measures, reaching 19.2% for the first and 20.8% for the latter. It is interesting to note how the familial link is predominant also after the reduction of containment measures after the summer period, which coincided with the reopening of schools, most work activities and public or private meeting places. We did not find any significance for all the variables and trends through categories.

**Table 4:** Trend of NST (positive for SARS-CoV2) divided for Epidemiological Link Clusters by Lockdown Phases

	Positive NST trend: Epidemiological Link by Lockdown Phases Lockdown Phases					
		National Lockdown	Phase 2	Phase 3	Phase 4	New Containment Measures
**Epidemiological Link Clusters**	**Familial** (N)	11 (91.7%)	5 (100%)	19 (79.2%)	104 (69.3%)	75 (60%)
	**Extra-Familial** (N)	1 (8.3%)	0 (0%)	4 (16.7%)	26 (17.3%)	24 (19.2%)
	**Unknown** (N)	0 (0%)	0 (0%)	1 (4.2%)	20 (13.3%)	26 (20.8%)
	**Total**	12 (3.8%)	5 (1.6%)	24 (7.6%)	150 (47.5%)	125 (39.6%)

*p* = NS (o > 0.05) for all the variables and trends through categories (ANOVA with post HOC analysis)

### Clinical manifestations and outcome

Clinical manifestations divided for age groups were summarized in Table 5. Fever was considered as temperature⥸ 37.5 °C and it was the most common clinical sign (66% of the total children positive for SARS-CoV2 infection), accounting for the highest percentage in the group of 0–6 years (71.9%). It was followed by upper airways symptoms such as rhinitis, rhinorrhea, pharyngitis, pharyngodynia, ear pain, external otitis, occasional cough (46%) and gastrointestinal symptoms such as diarrhea, vomiting and abdominal pain (11%). Loss of smell (or anosmia) and loss of taste (or ageusia) were reported only in older children and young adults with a percentage of 8.7% among children aged 11–15 years and 15.4% among patients aged 16–18 years.

**Table 5:** Clinical manifestations of children positive for SARS-CoV2 infection divided for each age group

***Clinical manifestasions***	***Group Age***				
	0–6 years	7–10 years	11–15 years	16–18 years	Total
**No Symptoms**	50	19	14	3	86
**Fever**	123	18	49	18	208
**Upper airways**	92	13	32	7	144
**Lower airways**	6	2	15	10	33
**Anosmia/Ageusia**	0	0	7	5	12
**Gastrointestinal symptoms**	19	4	7	4	34
**Other symptoms**	4	1	12	2	19

*p* = NS (o > 0.05) for all the variables and trends through categories (ANOVA with post HOC analysis)

Other symptoms including neurological symptoms (headache, syncope, convulsion), musculoskeletal symptoms (myalgia, arthralgia), cutaneous signs (skin rash, hives) were more frequently documented in older children from 11 to 18 years old (15 and 7.7% in the groups aged 11–15 years and 16–18 years respectively).

Older children aged 11–18 years were more frequently symptomatic than younger children, especially involving lower airways, with symptoms such as dyspnea, productive/dry hacking cough and chest pain. On the other hand, younger children aged 0–10 years had the major percentage of asymptomatic (29.2% among children aged 0–6 years, 48.7% among children aged 7–10 years) and upper airways symptoms. No significant differences were found.

Clinical manifestations and management of children positive for SARS-CoV2 were then analyzed.

The total cohort of children included 8.5% of patients with comorbidities such as prematurity, rheumatic disease, infantile cerebral palsy, trisomy 21, congenital heart disease, adrenoleukodystrophy, Bardet Biedl syndrome, endocrinological diseases (hypothyroidism, type 1 diabetes, adrenogenital syndrome) and 4.4% of infants < 1 months of age, with no significant difference for this variable. Considering each phase, the highest percentage of imaging (100%) and laboratory tests (80%) associated with a subsequent hospitalization (100%) was phase 2, although not significant. An interesting observation is the statistically significant decrease (*p* < 0.05) in performing chest-X ray with the progression to different phases and the concurrent increase in abdomen Ultrasound. The hospitalization showed a reduction from the first lockdown phase (91.7%) to the phase 4 (40.7%). ICU admissions represented 1% of the total hospitalizations. The two patients requiring intensive care were a female patient 16-years-aged presenting in coma state with diffuse-interstitial-pneumonia and a male patient 16-years-aged with dyspnea and severe pneumothorax. They were both without comorbidities and with unknown epidemiological links.

## Discussion

In this retrospective study we described a numerous cohort of 316 pediatric patients with SARS-CoV2 infection, admitted to our Pediatric Emergency Department from March 17th, 2020 to December 1st, 2020.

For the first time, we described changes in trend of infection during the principal Lockdown phases identified by the Italian Government during the pandemic and we evaluated epidemiological links and main clinical features among these historical periods, in order to demonstrate the efficacy of Italian Containment Measures before the introduction of the first COVID-19 vaccine in Italy on December 27th, 2020.

Over the period starting from March 17th, 2020 until December 1st, 2020 on a total of 13.703 admissions to our PED, 5001 children have been tested for SARS-CoV2 (36.5%). During the phase 4 we performed the highest percentage of NST 49.7%, and the highest percentage of positive NST on total NST performed 7.9%.

As already pointed out in Northern Italy and other country’s EDs and PEDs [16, 20–22], also our PED showed a marked decrease in the total number of accesses, due probably to Government recommendation to avoid direct access to ED and the general fear of becoming infected by accessing hospital facilities (https://flunewseurope.org/SeasonOverview).

Moreover, the introduction of preventing measures for the diffusion of SARS-CoV2 contributed to the decrease of other viruses circulation, as showed for influenza virus [23].

The age group with the highest number of positive NST is the group between 0 and 6 years. The group of 16–18 years old, including young adults, has the lowest finding of positive NST, but as a pediatric hospital, the majority of admissions are under 11 years old.

Interestingly, the high percentage of NST performed during the phase 4 could be attributed to the increased request of NST for the readmission at school or after a contact with a positive case of a family member, as registered in our electronic database.

Previously published studies have not shown complete concordance on the most common epidemiological link. Parri et al. described a large Italian cohort of 170 patients with SARS-CoV2 infection [3]. .They reported a 41% of patients with a familial relative positive at NST for SARS-CoV2, 42% reported a suspected extrafamilial contact, 12% travelled in endemic areas and 4% were symptomatic without a clear contact with a positive or suspect case. According to the author this finding is different from what has been observed in previous Chinese cohorts [2, 24, 25].

Garazzino et al., reported, in a cohort of 759 Italian patients, that 70.5% of children had at least one infected parent and 10% had an infected household [4].

In our study familial link was predominant during all phases of Lockdown in all age categories with 214 children on the total of 316 (68%), with a predominance during the first lockdown and phase 2. It is noteworthy how the familial link is predominant also after the reduction of containment measures during the summer period, which coincided with the reopening of schools, most work activities and public or private meeting places.

As reported by Romani et al. [26] in their cohort 38 out of 43 children belonged to a family cluster. Noteworthy in 37% of cases, they found that the family member was a healthcare worker.

We observed an increase of Extra-Familial and Unknown Link during the last phases, especially among older children and adolescents. The extra-familial link accounted for the highest percentage in the 11–15 years group (40%) and in the phase 4, coherent with the hypothesis that adolescents were infected mostly by peers during the summer.

About the intra-scholastic link, places where social distancing and the correct use of personal protective equipment (PPE) are observed, such as primary schools, the risk of being infected seems to be low, at least starting from 6 years of age [27]. In Italy more than 65,000 schools reopened in September, but only 1212 had experienced outbreaks about 4 weeks later and only one secondary school had a cluster of more than 10 infected people [28].

A prospective study on the prevalence of SARS-CoV2 infection among students and the association between the increase in transmissibility and the dates of reopening of schools in different regions, did not find a significant association between the reopening of schools and the increase of infection in the general population [29]. These findings have been supported by other case series in other states, all supporting a low risk in transmitting the infection in schools in Germany, Australia and England [30–32]. A common limit in these studies is the impossibility of distinguishing transmissions in schools and those attributable to the use of public transport, leisure time activities or sports [33].

In our cohort we found 17 (5.3% of the total cohort) patients with a clear intra scholastic exposure, detected from October 8th, 2020 to December 1st, 2020. Eight of them belonged to the 0–6 years age group 0–6 years, 5 to the 7–10 years age group, 3 to the 11–15 years age group, 1 to the 16–18 years age group. We remember nevertheless that high school and university remained closed since the Phase 1 of National Lockdown. Additionally, 29.5% referred to be infected by their teacher and were all aged 0–7 years, while the other 79.5% declared to be infected by classmates. These preliminary data about the intra-scholastic link suggest the need of further studies.

Parri et al. [3] observed that children were frequently categorized as asymptomatic (17%) or mild (63%), according to the classification of disease made by Dong et al. [2], differently from the previous Chinese cohorts [24, 25], in which it was reported a majority of moderate cases. Garazzino et al. observed a 12% of asymptomatic children and the majority showed mild symptoms such as cough or rhinitis [4]. Gotzinger et al. reported in a multicenter multinational cohort study involving 582 individuals, a 16% of asymptomatic patients [34].

In our study we have found a milder clinical presentation of children admitted to PED according to different age groups.

Previous studies reported fever respectively in 48% of children according to Parri et al. [3]; 41% according to Lu et al. [24] and 36% according to Qiu et al. [25] while Garazzino et al. reported fever as the most common symptom among their pediatric cohort (81.9%), especially among infants [4].

We also observed a high frequency of fever (66%), in particular among preschooler children aged 0–6 years (71.9%) in our study, and it could be explained by the fact that they analyzed patients in a period ranging from March 3rd to May 2nd, 2020 which coincided with our lockdown phase and initial phase 2, when the implementation of medical phone consultation, telemedicine, and tele-expertise was encouraged, especially for febrile patients. Indeed, we found the highest percentage of symptom fever in Phase 1, 79% and Phase 2.67%. We used 37.5 °C as limit value to consider febrile patients with SARS-Cov2 according to the previous literature about SARS-Cov2 infection in pediatric population [3, 4, 24] and used this limit to perform NST in patients admitted to our PED according to the Italian Government guidelines [11].

As mentioned above the decision to test patients for SARS-CoV2 varied frequently among the different phases of lockdown. During early phases we tested mainly children with respiratory symptoms or fever, close contacts with a proven COVID-19 subject, or recent travel to specific endemic areas listed and constantly updated by WHO [35]. During last phases we started performing NST for SARS-CoV2 on patients with gastrointestinal symptoms, according to recent literature reporting gastrointestinal symptoms and associated complications, as symptoms associated with SARS-CoV2 infection [4, 36, 37]. Probably for this reason we detected a progressive occurrence of gastrointestinal symptoms in infected patients during the last period of observation (Phase 3 12.5%, Phase 4 10% and New containment Measures 12.8%) with a total of 34. Six abdomen UltraSound were performed (1.9%) and all of them in the New Containment Measures phase (Table 4).

Loss of smell and the loss of taste were typical symptoms of COVID-19 that we reported only in older children in line with other previous studies. The categories of older children aged 11–18 years also resulted more frequently symptomatic than younger children with two 16-years-old patients requiring ICU admissions from the PED. The reason for this phenomenon could be that parents of asymptomatic or pauci-symptomatic older children did not bring them to our PED, preferring to manage mild clinical symptoms at home, according to the recommendations of the Italian Ministry of Health. For this reason, it’s mandatory to focus on adolescents and young adults that came to PED with moderate/severe symptoms requiring in severe cases intensive care.

Other “special categories” include children with comorbidities (the most common were respiratory, cardiac or neuromuscular chronic diseases). These categories needed frequent hospitalization and, in some cases, respiratory support, or even treatment in the Intensive Care Unit. Infants aged < 1 month represented about 12.9% of the total cohort of children (*p* > 0,05).

In our opinion and according to other studies [3, 4, 34] involving pediatric cases, these “special needs” categories have to be focused on because of the risk of rapid clinical deterioration and the high rate of hospitalization.

Dong and colleagues reported that, on a sample of 2143 pediatric patients, the proportion of severe and critical cases was 10.6, 7.3, 4.2, 4.1 and 3.0% for the age group of <1, 1–5, 6–10, 11–15 and > 15 years, but they analyzed only the period from January 16, 2020, to February 8, 2020. The limit of the study was that only 731 patients (34%) were laboratory-confirmed, while the others had only a clinical diagnosis based on respiratory symptoms [2].

While in Parri et al. study [3] 36% performed a Chest X ray and among them 52% showed some alterations such as interstitial abnormality or consolidations, in our cohort we have performed 158 Chest X-ray (50%), with a prevalence of interstitial pattern or consolidations (*p* < 0,05). We found a progressive reduction in number of Chest X-ray performed during last period, this could probably be attributed to the increase in the expertise of our practitioner in detect patients who requires an imaging, thanks to the better knowledge of respiratory complications in pediatric patients.

Blood tests (including blood cell count and chemistry routine) were performed on 32,3% of children (*p* > 0,05).

We observed a total of 63% of patients needing hospitalization during all phases of lockdown, in line with previous literature [4, 34]. We reported in particular a significant decrease from the first lockdown phase, 91.7% to the phase 4, 40.7% (*p* < 0,05). This data was congruent with the finding of patients more asymptomatic (19%) or pauci-symptomatic in the last phase than in the first phase (asymptomatic patients were 0%), due to the increase in the number of NST performed and to the tendency to hospitalize only patients at high risk, with comorbidities or with a moderate-severe symptom. We reported 2 patients needing ICU admission, both 16-years old. Gotzinger et al. reported 48 patients requiring ICU admission and significant risk factors were being younger than 1-month, male sex, comorbidities and lower respiratory tract infections [34].

Pediatricians working in the PED should pay attention not only to patients with acute infection and a positive NST for SARS-CoV2 but also to those patients less than 21 years of age presenting with persistent fever > 38 °C for at least 24 h, with multisystem (> 2) organ involvement (cardiac, renal, respiratory, hematologic, gastrointestinal, dermatologic or neurological) laboratory evidence of inflammation and the history of recent SARS-CoV-2 infection by RT-PCR, serology, or antigen test; or COVID-19 exposure within the 4 weeks prior to the onset of symptoms, possibly presenting MIS-C [38].

We have observed 7 patients with a final diagnosis of MIS-C, they were all admitted during the last phase of New Containment Measures, the mean age of our patients was 8.5 years-old with an interquartile age of 1.8–12.8.

### Limitations of the study

All patients were enrolled from a single PED, situated in Rome, Lazio, Italy, an area partially spared in the early stages of the pandemic. The Age Groups were not homogeneous in number of patients, in particular for the 16–18 years old group, because the total admissions, as a Pediatric hospital, for this specific group were normally low. Our hospital is a COVID-19 Hub for numerous peripheric hospital, so an important number of patients were hospitalized without being admitted in PED. Data have been collected by different operators, even with a standardized method. Parents and caregivers of a tested positive patients in our PED, were not routinely tested with a NST, unless the patients were hospitalized.

## Conclusions

Our study covered a long observational period and enrolled the largest cohort of Italian pediatric patients, admitted to a single PED, with SARS-CoV2 infection. Different levels of containment measures caused important changes in number of positive NST for SARS-CoV2. Our data supported the efficacy of Italian Containment Measures. Familial link was predominant in our cohort, during all phases of Lockdown. The risk of being infected at home is four time greater than the risk of being infected from an extra familial individual, as already reported in previous literature. Further studies, involving large number of patients, are needed to evaluate the clear impact of intra-scholastic link. The constant improvement in knowledge on onset symptoms and risk factor for SARS-CoV2 infection and its complications (e.g. MIS-C), can impact on number of hospitalizations, ICU admissions and early management.

## Data Availability

All data have been collected from Electronic Registers. The datasets used and analysed during the current study are available from the corresponding author on reasonable request.

## Abbreviations

NST: Nasopharyngeal Swab Test
ICU: Intensive Care Unit
SARS-CoV2: Severe Acute Respiratory Syndrome- CoronaVirus 2
COVID-19: Coronavirus Disease 19
ARDS: Severe acute respiratory distress syndrome
MOF: Multi-organ failure
WHO: World Health Organization
PED: Pediatric Emergency Department
CDC: Center for Disease Control and Prevention
RT-PCR: Real Time- Polymerase Chain Reaction
PPE: Personal protective equipment
